# The Involvement of TRP Channels in Bone Homeostasis

**DOI:** 10.3389/fendo.2012.00099

**Published:** 2012-08-20

**Authors:** Liesbet Lieben, Geert Carmeliet

**Affiliations:** ^1^Clinical and Experimental Endocrinology, Department of Clinical and Experimental Medicine, Katholieke Universiteit LeuvenLeuven, Belgium

**Keywords:** bone, TRP channels, calcium, calcium channels, osteoblast, osteoclast, intestine, kidney

## Abstract

Calcium and bone homeostasis are intimately related. On the one hand, bone relies on a sufficient supply of calcium to maintain its structural and mechanical properties and thus largely depends on calcium absorption in the intestine and calcium reabsorption in the kidney. On the other hand, bone serves as a calcium reserve from which calcium is mobilized to maintain normal calcium levels in blood. A negative external calcium balance will therefore at all times impair skeletal integrity. In addition to the external calcium balance, skeletal homeostasis also depends on the proper differentiation and functioning of bone cells, which relies for a large part on intracellular Ca^2+^ signaling. Members of the transient receptor potential (TRP) family of ion channels affect skeletal homeostasis by mediating processes involved in the extracellular as well as intracellular Ca^2+^ balance, including intestinal calcium absorption (TRPV6), renal calcium reabsorption (TRPV5), and differentiation of osteoclasts (TRPV1, TRPV2, TRPV4, TRPV5), chondrocytes (TRPV4), and possibly osteoblasts (TRPV1). In this review, we will give a brief overview of the systemic calcium homeostasis and the intracellular Ca^2+^ signaling in bone cells with special focus on the TRP channels involved in these processes.

## Introduction

Calcium is crucial for bone homeostasis, as the skeleton is influenced both by the external Ca^2+^ balance as well as by the intracellular Ca^2+^ signaling in bone cells. First of all, the proper functioning of the skeleton relies on normal serum calcium levels, but bone also plays an important role in the maintenance of systemic calcium homeostasis. Indeed, 99% of the bodily calcium is stored in bone where it contributes to its mechanical and structural properties. Consequently, bone requires a sufficient supply of calcium to maintain skeletal integrity. This calcium supply mainly depends on calcium absorption in the intestine and calcium reabsorption in the kidney. A failure of one of these processes, as may occur during insufficient dietary calcium intake, aging, and gastrointestinal/renal disorders, therefore greatly increases the risk of a calcium imbalance and (osteoporotic) bone loss (Bullamore et al., 1970; Bikle, 2007). These disorders will not only reduce the calcium supply to bone but may also actively reduce the calcium content in bone as the safeguarding of normal serum Ca^2+^ levels will always predominate over the calcium storage in bone. Consequently, bone will contribute significantly to the external calcium balance by displacing calcium to serum. Although this system is crucial to maintain normal serum Ca^2+^ levels and thus to guarantee the optimal functioning of multiple vital processes, the effect on bone strength is devastating with an increased fracture risk as a consequence.

Secondly, intracellular Ca^2+^ is also an important second messenger in bone cells. Intracellular Ca^2+^ signaling in osteoblasts, osteoclasts, chondrocytes, and nerve endings has been shown to regulate numerous functions, including differentiation, signal transduction, and sensing of mechanical, osmotic, and pain stimuli. The fine-tuning of the intracellular Ca^2+^ levels is thus crucial for normal bone homeostasis, and abnormalities in the transporters involved in Ca^2+^ signaling will unambiguously lead to diseases that also affect bone structure or function (Blair et al., 2007).

**Figure 1:**
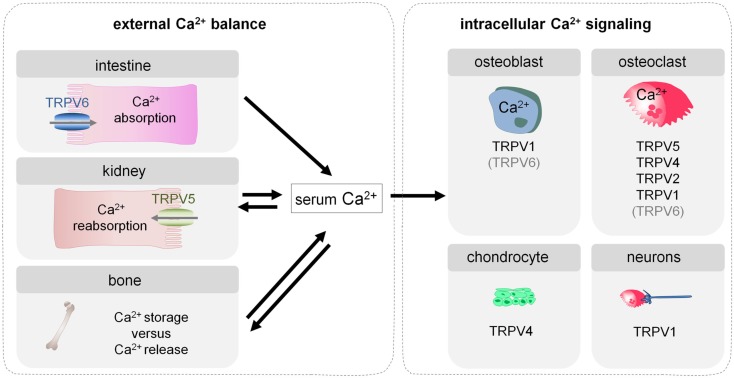
**Involvement of TRP channels in the external calcium balance and intracellular Ca^2+^ signaling in bone cells**. Bone homeostasis depends on the external calcium balance which determines whether calcium is stored in or released from bone, and consequently affects skeletal strength. In addition, bone homeostasis is also influenced by intracellular Ca^2+^ signaling in bone cells, which regulates their differentiation and functioning. Several TRP channels contribute to both types of processes and more specifically modulate intestinal calcium transport (TRPV6), renal calcium reabsorption (TRPV5), and differentiation and functioning of osteoblasts (TRPV1), osteoclasts (TRPV5, TRPV4, TRPV2, TRPV1), and chondrocytes (TRPV4). Moreover, TRPV1 expressed in the neurons that innervate bone, plays a crucial role in the sensing of bone pain.

The maintenance of extra- and intracellular Ca^2+^ homeostasis is thus crucial for bone biology, and depends for a large part on Ca^2+^ channels. Several types of Ca^2+^ channels exist including (i) ryanodine receptors (RyR) and inositol-1,4,5-trisphosphate receptors (IP_3_R), which mediate the release of Ca^2+^ from the endoplasmic reticulum (ER), (ii) store-operated calcium channels (SOCE), which include ORAI1 and STIM1 and which mediate the flux of extracellular Ca^2+^ into the ER via ORAI1 upon intracellular store depletion sensed by STIM1, (iii) voltage-gated Ca^2+^ channels (VGCC) that allow Ca^2+^ influx upon cell depolarization, (iv) stretch-activated Ca^2+^ channels that mediate Ca^2+^ influx after mechanical stimulation, and (v) the transient receptor potential (TRP) family of cation channels (Robinson et al., 2010). In this review, we will focus on the involvement of the TRP channels. The TRP family consists of 28 members which have diverse physiological functions. They are subdivided based on their sequence into six subfamilies, i.e., TRPC (canonical), TRPV (vanilloid), TRPP (polycystin), TRPM (melastatin), TRPA (ankyrin), and TRPML (mucolipin). Especially the members of the TRPV family of ion channels are involved in the extracellular calcium homeostasis and intracellular Ca^2+^ signaling in bone cells. The TRPV family consists of six members, which are all membrane proteins composed of six transmembrane domains that form a cation-permeable pore region. TRPV1–4 are non-selective cation channels, whereas TRPV5 and six are highly Ca^2+^ selective. TRPV channels are activated by a variety of stimuli. TRPV1 is activated by heat, noxious stimuli, a low pH, and numerous chemicals. TRPV2–4 share their activation by heat albeit at different thresholds. Moreover, TRPV2 and 4 are activated by hypotonicity. TRPV1–4 are therefore involved in nociception, thermo-, and mechanosensing. TRPV5 and 6 are different from the other TRPV channels in that they are highly selective to Ca^2+^ and that they are not activated by heat but by low intracellular Ca^2+^ levels. An extensive overview of the structural and functional aspects of the different members of the TRP family is beyond the scope of this manuscript, but can be found in the following articles (Hoenderop et al., 2005; Pedersen et al., 2005; Nilius et al., 2007). We will focus on the TRP channels that are involved in the maintenance of the external calcium balance (TRPV5, TRPV6) and contribute to intracellular Ca^2+^ signaling in bone cells (TRPV5, TRPV4, TRPV2, TRPV1; Figure 1). We will first give a general overview of calcium homeostasis (see Regulation of the External Calcium Balance and its Repercussion on Bone Homeostasis) followed by a synopsis on intracellular Ca^2+^ signaling in bone cells (see Intracellular Ca^2+^ Signaling in Bone Cells). In each of these two parts we mention the most important Ca^2+^ channels and briefly describe the TRPV channels involved. Finally, we will discuss in more detail the functions of each of the specific TRPV channels in the different tissues and cell types that have a role in calcium homeostasis and bone biology (see Involvement of TRP Channels in Bone Biology).

## Regulation of the External Calcium Balance and Its Repercussion on Bone Homeostasis

**Figure 2:**
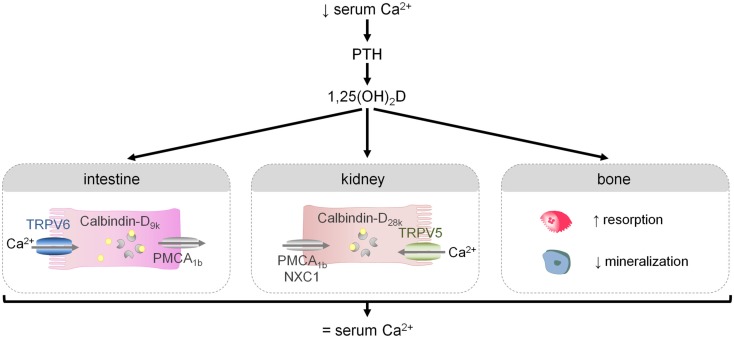
**The regulation of the external calcium balance**. A reduction in serum Ca^2+^ levels triggers the release of PTH and the production of 1,25(OH)_2_D, the active hormone. 1,25(OH)_2_D in turn modulates several processes that will ultimately lead to normalization of serum Ca^2+^ levels: it will increase intestinal and renal calcium transport, and when these adaptations are insufficient, 1,25(OH)_2_D will mobilize calcium from bone by stimulating osteoclastic bone resorption and by inhibiting bone mineralization.

The regulation of systemic calcium homeostasis is directed at maintaining serum Ca^2+^ levels within a very narrow range. Normocalcemia is achieved by a complex endocrine system that modulates calcium absorption in the intestine, net calcium secretion in the kidney, and calcium deposition/mobilization from bone. Briefly, small changes in blood Ca^2+^ levels trigger the secretion of parathyroid hormone (PTH) from the parathyroid gland. PTH, in turn, promotes the renal formation of active vitamin D, 1,25(OH)_2_D, from its inactive precursor. 1,25(OH)_2_D action is key to ensure normocalcemia by regulating processes in the different calcemic target tissues. Indeed, 1,25(OH)_2_D enhances intestinal calcium transport and renal calcium reabsorption. When these adaptations are insufficient to restore normocalcemia, 1,25(OH)_2_D triggers a shift of calcium from bone to serum. Further activation of this endocrine control system is suppressed by the normalization of serum Ca^2+^ levels and by the several negative feedback loops (Figure 2; Bouillon et al., 2008; Lieben et al., 2011).

Since the diet is the only source of calcium, calcium absorption in the intestine is a crucial first step in maintaining systemic calcium homeostasis. Intestinal calcium transport consists of two pathways: a passive, paracellular mechanism that depends on high dietary calcium levels, and an active pathway that predominates when calcium intake is normal/low and in particular this mechanism is stimulated by 1,25(OH)_2_D. Active calcium transport is considered to involve a transcellular mechanism with calcium entry through an apical Ca^2+^ channel, followed by intracellular calcium binding to members of the Calbindin family (Calbindin-D_9k_) and extrusion to the extracellular milieu by the Ca^2+^-ATPase, PMCA_1b_ (Figure 2; Van Cromphaut et al., 2001). One member of the TRP family, namely TRPV6, has long been considered to be the rate-limiting step in the active transport of calcium across the enterocyte by mediating apical Ca^2+^ transfer (Hoenderop et al., 2005). Recent genetic studies confirm that TRPV6 contributes to active intestinal calcium transport, but they also indicate that this channel is not essential for this process (Benn et al., 2008). A detailed overview of the role of TRPV6 in intestinal calcium transport and bone homeostasis will be given in Section “TRPV6.”

Fine-tuning of renal calcium reabsorption also contributes to the maintenance of normocalcemia. Half of the unbound serum Ca^2+^ is intrinsically filtered in the glomerulus, the bulk of this filtered Ca^2+^ is passively reabsorbed in the nephron. Reabsorption of the remaining 15% involves an active transport pathway, that resembles intestinal calcium transport but relies on other mediators, i.e., calcium entry via TRPV5, cytosolic binding to Calbindin-D_28k_ and extrusion by the Na^+^/Ca^2+^ exchanger (NCX1) and PMCA_1b_ (Figure 2; Bouillon et al., 2008). In contrast to the limited role of TRPV6 in intestinal calcium transport, genetic evidence has confirmed that TRPV5 is the essential and rate-limiting step in renal calcium reabsorption (Hoenderop et al., 2003), which will be discussed in more detail in Section “TRPV5.”

Calcium stored in bone has two important functions, i.e., providing mechanical strength and acting as a calcium reserve. The inherent consequence of this dual role is that it will be difficult, or even impossible to fulfill these two functions when the external calcium balance is negative and experimental evidence indicates that it is the maintenance of normocalcemia that will be secured by all means. Consequently, a negative calcium balance will lead to bone loss, which hampers the mechanical function of the skeleton and may result in an increased fracture risk. We recently showed that in mice, increased 1,25(OH)_2_D levels play a crucial role in this shift of calcium from bone to serum that occurs during a negative calcium balance. Mechanistically, increased 1,25(OH)_2_D levels enhance osteoclastic bone resorption and suppress bone matrix mineralization by increasing the osteoblastic expression of mineralization inhibitors including Osteopontin and pyrophosphates (Figure 2; Lieben et al., 2012). The preservation of normal calcium levels in blood and bone is thus closely interconnected, and this situation also implies that the skeleton not only depends on, but moreover participates in the external calcium balance. It is therefore evident that abnormalities in the external calcium balance due to loss of TRPV5 (and TRPV6) will also affect bone homeostasis (see TRPV6 and TRPV5).

## Intracellular Ca^2+^ Signaling in Bone Cells

Normal bone homeostasis does not only depend on extracellular calcium, but also on intracellular Ca^2+^ signaling cascades that regulate the differentiation and functioning of multiple bone cells. In the next section, we will give a concise overview of the molecules involved in intracellular Ca^2+^ signaling in the bone resorbing osteoclasts (see Ca^2+^ Signaling in Osteoclasts), the bone forming osteoblasts (see Ca^2+^ Signaling in Osteoblasts), and the chondrocytes which contribute to endochondral bone development and form the articular cartilage (see Ca^2+^ Signaling in Chondrocytes).

### Ca^2+^ signaling in osteoclasts

Recent genetic studies have provided substantial insight in the molecular mechanisms that are involved in and regulate osteoclastic Ca^2+^ signaling and differentiation. In short, binding of macrophage-colony stimulating factor (M-CSF) to its receptor on osteoclasts precursors promotes their proliferation and survival and induces receptor activator of nuclear factor-κß (RANK) expression (Negishi-Koga and Takayanagi, 2009). Osteoclast differentiation is initiated by the simultaneous activation of RANK after binding to RANK-ligand (RANKL), and of the immunoreceptor tyrosine-based activation motif (ITAM)-associated immunoglobulin-like receptor (IgLR; Takayanagi et al., 2002; Koga et al., 2004). These signaling pathways induce the activation of phospholipase Cγ (PLCγ). PLCγ produces IP_3_, which evokes Ca^2+^ release from the ER via IP_3_ receptors, that will subsequently lead to typical Ca^2+^ oscillations (Kuroda et al., 2008). *In vitro* findings suggest that TRP channels (TRPV2, see TRPV2; Kajiya et al., 2010) are likely one of the Ca^2+^ entry pathways that contribute to the Ca^2+^ oscillations (Kajiya et al., 2010; Hwang and Putney, 2011). With respect to the role of the SOCE, the reported findings are still limited. In this regard, *in vivo* inactivation of *Orai1* in mice is reported to impair the formation of multinucleated osteoclasts and to reduce bone resorption. Consistent herewith, *in vitro* inhibition of *Orai1* in a murine monocyte/macrophage cell line (chemical and siRNA) decreased osteoclastogenesis (Hwang and Putney, 2011; Robinson et al., 2012). Yet conclusive genetic confirmation based on the generation of osteoclast-specific *Orai1* null mice is still lacking and the contribution of ORAI1 to Ca^2+^ signaling during osteoclast differentiation remains elusive. No VGCC’s have been detected thus far in osteoclasts, implying that it is unlikely that they play a role in osteoclast differentiation (Blair et al., 2007).

**Figure 3:**
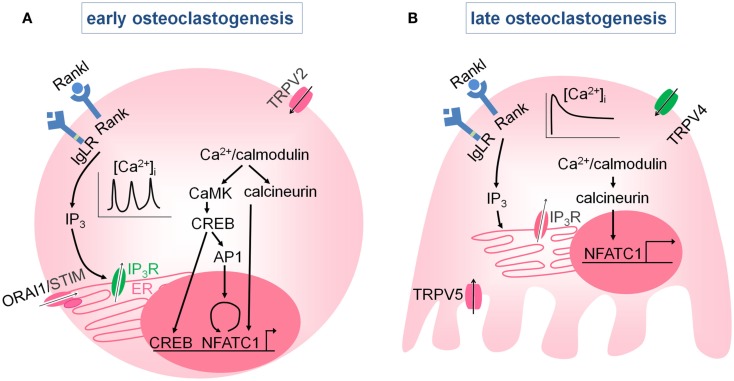
**Intracellular Ca^2+^ signaling in osteoclasts**. **(A)** Early during osteoclast differentiation, activation of the RANK, and IgLR receptors induces intracellular Ca^2+^ oscillations by intracellular Ca^2+^ release via the IP_3_R, and possibly via Ca^2+^ influx through store-operated Ca^2+^ channels or TRP (TRPV2) channels. The Ca^2+^ oscillations activate Ca^2+^/ calmodulin-dependent kinases (CaMK, calcineurin) which induce autoamplification of *Nfatc1* and NFATc1-mediated gene transcription. **(B)** At later stages, the Ca^2+^ oscillations diminish and are replaced by a sustained Ca^2+^ influx that is predominantly mediated by TRPV4, although TRPV5 may also play role. Both the Ca^2+^ oscillations and the sustained Ca^2+^ influx are required for the NFATc1-mediated stimulation of osteoclast differentiation.

The Ca^2+^ oscillations turn on a number of Ca^2+^/calmodulin-activated proteins including calcineurin and calmodulin-dependent protein kinases (CaMK). Upon activation of the phosphatase calcineurin, the transcription factor NFATc1 (the nuclear factor of activated T cells c1) becomes phosphorylated, translocates to the nucleus, and increases osteoclast-specific gene transcription. NFATc1 is the master regulator of osteoclast differentiation, evidenced by the complete absence of osteoclasts in conditional *Nfatc1^−/−^* mice (Aliprantis et al., 2008). Ca^2+^/calmodulin signaling also activates the CaMK-mediated CREB (cAMP response element-binding protein) pathway, which increases in cooperation with NFATc1, the osteoclast-specific gene expression. In addition, CREB induces cFOS in the AP (activator protein) 1 complex, which contributes to the autoamplification of *Nfatc1* (Sato et al., 2006). The RANK- and IgLR-induced Ca^2+^ oscillations are thus crucial in the initiation of osteoclastogenesis by promoting NFATc1 and CREB activity (Figure 3A).

Ca^2+^ oscillations disappear during osteoclast differentiation and are replaced by a sustained Ca^2+^ influx via members of the TRP family, including TRPV4 (see TRPV4; Masuyama et al., 2008) and possibly TRPV5 (see TRPV5; Chamoux et al., 2010; Figure 3B). The Ca^2+^ oscillations followed by the sustained Ca^2+^ influx are both needed for NFATc1 activation and proper osteoclast differentiation. For more in debt information, we refer to the review article by Negishi-Koga and Takayanagi (2009).

### Ca^2+^ signaling in osteoblasts

**Figure 4:**
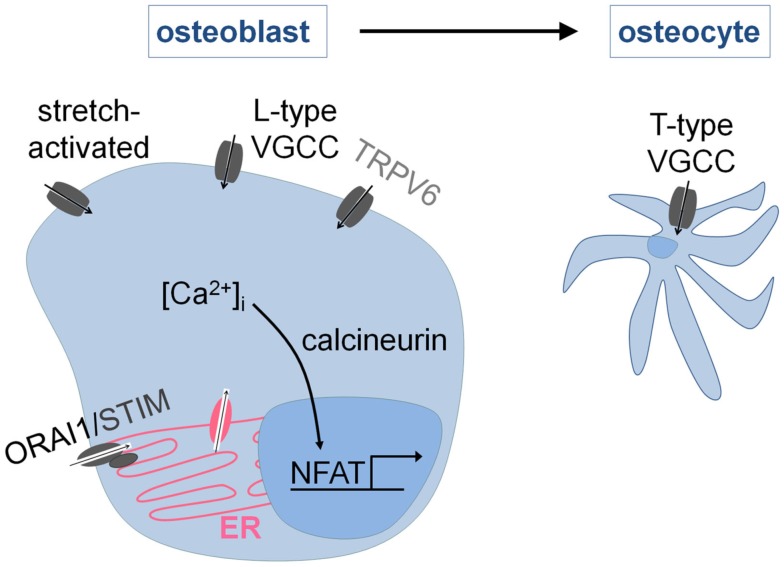
**Intracellular Ca^2+^ signaling in osteoblasts**. Osteoblasts express stretch-activated, voltage-gated (VGCC), store-operated (ORAI1), and TRP (TRPV6) Ca^2+^ channels. The response to mechanical stimuli involves Ca^2+^ entry via L-type (osteoblasts) and T-type (osteocytes) VGCC’s. In addition, it is known that osteoblast differentiation requires the activation of NFAT-mediated gene transcription by Ca^2+^/calcineurin. The Ca^2+^ entry pathways are incompletely characterized but ORAI1 is likely involved.

In contrast to the osteoclasts, little is known about the molecular mechanisms mediating osteoblastic Ca^2+^ signaling (Blair et al., 2007; Figure 4). Osteoblasts express different families of Ca^2+^ channels, including members of the store-operated (ORAI1), the stretch-activated, the voltage-gated, and the TRP family (TRPV6, see TRPV6) of calcium channels. VGCC’s are important for proper osteoblast functioning and more specifically for the propagation of calcium waves across neighboring osteoblasts upon mechanical stimulation. Recent *in vitro* studies have demonstrated that the sensitivity and the dynamics of the calcium waves are even greater in finally differentiated osteoblasts, i.e., osteocytes, which are believed to be the true mechanosensors of bone. The changes in the calcium waves with differentiation are attributed to the presence of a different subset of VGCC’s, i.e., L-type VGCC’s in osteoblasts versus the T-type VGCC’s in osteocytes (Lu et al., 2012).

In addition, Ca^2+^ signaling is very plausibly involved in osteoblast differentiation. Chemical inhibition of calcineurin activity causes a decrease in bone mass and acts by impairing bone formation (Koga et al., 2005). Similar results are obtained when the calcineurin Aα isoform is genetically inactivated (Sun et al., 2005). In contrast, osteoblast-specific inactivation of the calcineurin B subunit stimulates bone formation (Yeo et al., 2007), indicating that the role of calcineurin in osteoblast differentiation requires further investigation. The results regarding the role of NFAT signaling in osteoblasts, on the other hand, are more consistent, i.e., genetic inactivation of NFATc1 and NFATc2 in osteoblasts impairs osteoblastogenesis leading to osteopenia, whereas an opposite phenotype is observed when NFATc1 is specifically activated in the osteoblastic lineage (Koga et al., 2005; Winslow et al., 2006). The Ca^2+^ influx pathways that mediate calcineurin/NFAT activation in osteoblasts, however, are still largely unknown, and require further investigation. Recent data suggest that the store-operated Ca^2+^ channel ORAI1 is involved, as genetic inactivation of *Orai1* impairs osteoblast differentiation and bone formation *in vitro* and *in vivo* (Robinson et al., 2012).

### Ca^2+^ signaling in chondrocytes

**Figure 5:**
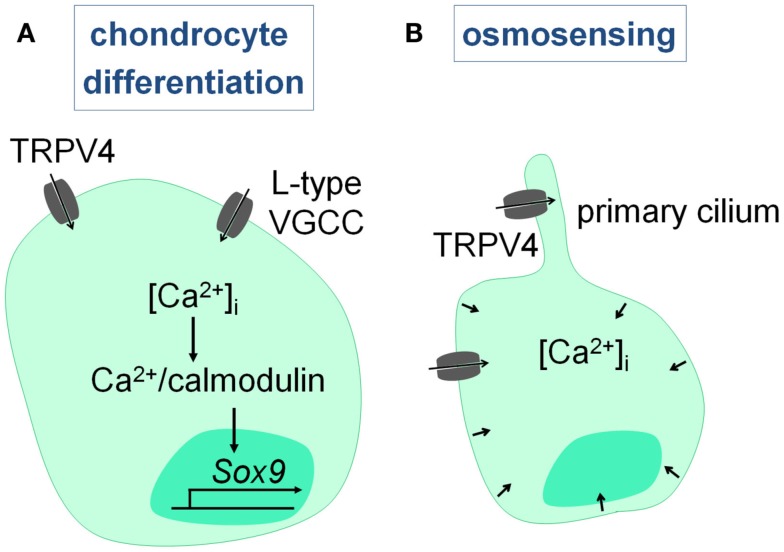
**Intracellular Ca^2+^ signaling in chondrocytes**. **(A)** Chondrocyte differentiation involves Ca^2+^ entry via L-type VGCC’s and TRPV4, that leads to the Ca^2+^/calmodulin-mediated induction of SOX9. **(B)** Hypo-osmotic stimuli activate TRPV4, and most likely the TRPV4 channels present on the primary cilium, leading to the increase in intracellular Ca^2+^ levels which is required for the volumetric changes and chondroprotection.

Chondrocytes express several Ca^2+^ channels, including VGCC’s, stretch-activated Ca^2+^ channels, and TRP channels (TRPV4), which are involved in chondrocyte differentiation and osmosensing. First of all, calcium influx via the L-type VGCC’s and TRPV4 is required to activate a Ca^2+^/calmodulin signaling cascade that promotes chondrocyte differentiation. Consequently, suppression of L-type VGCC’s (Mancilla et al., 2007), TRPV4 (Muramatsu et al., 2007), and Ca^2+^/calmodulin (Taschner et al., 2008) signaling hampers chondrocyte differentiation in rat metatarsal cultures, chondrogenic cell lines, and chicken chondrocytes respectively. Thus, Ca^2+^ influx via VGCC’s and TRPV4 (see TRPV4) is required for proper chondrogenesis (Figure 5A).

Secondly, Ca^2+^ signaling is involved in the osmosensing properties of chondrocytes, which are essential to maintain joint health. The articular chondrocytes in particular are exposed to large variations in their osmotic environment due to changes in the water content of the cartilage matrix upon loading and reloading of the joint. Chondrocytes respond directly to this osmotic stress by promoting Ca^2+^ influx and increasing intracellular Ca^2+^ levels which leads to volumetric changes. Abnormalities in this system result in or fasten the progression of rheumatoid arthritis. Recent studies have provided compelling evidence that TRPV4 is critically involved in the sensing of hypo-osmotic stimuli (Guilak et al., 2010), which we will further discuss in Section “TRPV4” (Figure 5B).

## Involvement of TRP Channels in Bone Biology

Transient receptor potential channels may affect skeletal homeostasis not only by regulating the external calcium balance but also by directly altering Ca^2+^ signaling in bone cells. The TRP channels that are known to be involved in one of these processes will be discussed in more detail (Figure 1).

### TRPV6

Active intestinal calcium transport is required when dietary calcium intake is normal/low. This pathway is stimulated by 1,25(OH)_2_D and involves calcium entry mediated in part by TRPV6, intracellular calcium binding to Calbindin-D_9k_, and basolateral extrusion of calcium via PMCA_1b_ (see Regulation of the External Calcium Balance and its Repercussion on Bone Homeostasis; Figure 2). TRPV6 has for long been considered to be the rate-limiting step in active intestinal calcium absorption, based on the finding that intestinal TRPV6 levels are altered in parallel with 1,25(OH)_2_D activity and inversely with dietary calcium intake. Indeed, *Trpv6* mRNA levels are upregulated by 1,25(OH)_2_D treatment and a low dietary calcium intake, whereas *Trpv6* expression is reduced in *Vdr* null mice or in response to a high calcium intake (Van Cromphaut et al., 2001).

Genetic studies confirm that TRPV6 is involved in intestinal calcium transport, but they also show that TRPV6 is not essential for this pathway. Indeed, active intestinal calcium transport is only minimally affected in *Trpv6* and *Trpv6/Calbindin-D_9k_* double null mice when mice are fed a diet containing a normal level of calcium (Bianco et al., 2007; Benn et al., 2008). In addition, 1,25(OH)_2_D treatment results in a similar increase in intestinal calcium transport in *Trpv6^−/−^*, *Trpv6/Calbindin-D_9k_^−/−^*, and wild-type mice (Benn et al., 2008; Kutuzova et al., 2008). Dietary calcium deprivation also stimulants intestinal calcium transport in all genotypes, yet the increase was less profound in the *Trpv6^−/−^* and *Trpv6/Calbindin-D_9k_^−/−^* mice (Bianco et al., 2007; Benn et al., 2008). Altogether, these results indicate that TRPV6 is redundant for intestinal calcium absorption during normal calcium intake, but that this Ca^2+^ channel does contribute to this process when dietary calcium levels are low.

Thus, TRPV6 contributes only minimally to intestinal calcium transport during normal calcium intake. Consequently, *Trpv6^−/−^* mice have normal serum calcium levels (Bianco et al., 2007; Benn et al., 2008) and display no abnormalities in bone homeostasis, evidenced by normal bone mass and remodeling parameters. Also the growth plate morphology is not affected which accords with normal serum calcium and phosphate levels (Lieben et al., 2010). Similarly, knock-in mice with a replacement of one amino acid of the *Trpv6* gene (*Trpv6^D541A/D541A^* mice) that impairs the Ca^2+^ permeability of TRPV6, do not display changes in bone mass and morphology, yet their bone size is slightly reduced (van der Eerden et al., 2012). In contrast to the redundancy of TRPV6 during normal calcium intake, TRPV6 is required for optimal intestinal calcium absorption when dietary calcium is limited. Accordingly, *Trpv6* null mice on a low calcium diet could maintain normal serum calcium levels, yet their bone matrix mineralization is more profoundly reduced compared to wild-type mice (Lieben et al., 2010). Thus, TRPV6 is essential to ensure adequate intestinal calcium absorption during dietary calcium deprivation, and hereby prevents an excessive decrease in bone mineralization. These findings also underscore the essential role of adequate dietary calcium absorption for skeletal health.

The absence of bone abnormalities during normal calcium intake indicates that TRPV6 only indirectly affects the skeleton via its involvement in intestinal calcium transport. Yet, TRPV6 is expressed in osteoblasts and osteoclasts, albeit at very low levels (1% of intestinal levels; Lieben et al., 2010; Little et al., 2011), and may thus in theory directly regulate their differentiation or functioning. Recent studies have however convincingly demonstrated that TRPV6 is not involved in osteoblastic Ca^2+^ uptake (Little et al., 2011) nor mineralization (van der Eerden et al., 2012). Altogether, these results confirm that TRPV6 lacks a direct role in bone metabolism (Figure 4).

In conclusion, TRPV6 affects skeletal metabolism by promoting intestinal calcium transport, and is especially required during dietary calcium deprivation.

### TRPV5

TRPV5 is critically involved in renal calcium reabsorption (see Regulation of the External Calcium Balance and its Repercussion on Bone Homeostasis; Figure 2). Indeed, inactivation of *Trpv5* results in a decrease in renal calcium reabsorption, leading to severe urinary calcium loss. Normal serum calcium levels are maintained via a compensatory, 1,25(OH)_2_D-mediated increase in intestinal calcium absorption. Furthermore, cortical bone mass is reduced in *Trpv5^−/−^* mice, which is associated with an increase in osteoclast number, though bone resorption parameters are reduced (Hoenderop et al., 2003). These results convincingly demonstrate that TRPV5 is important for systemic calcium homeostasis by fine-tuning renal calcium reabsorption.

The skeletal defect in *Trpv5^−/−^* mice may be due to the abnormal external calcium balance and/or to direct changes in osteoclastic Ca^2+^ signaling. Of note, TRPV5 is not expressed in osteoblasts. In osteoclasts, on the other hand, TRPV5 is found at the ruffled border, where it likely contributes to bone resorption. van der Eerden et al. reported that the *in vitro* differentiation of hematopoietic precursors isolated from *Trpv5* null mice leads to more and larger osteoclasts, but with severely impaired resorptive capacity. The resorption deficit is attributed to the lack of TRPV5 activity, whereas the enhanced osteoclastogenesis is explained by an increased priming of the osteoclast precursors by the high 1,25(OH)_2_D levels in *Trpv5^−/−^* null. These finding are in agreement with the increased osteoclast number but reduced bone resorption observed in *Trpv5* null mice, yet it is still unclear how these findings explain and correlate with the reduced bone mass *in vivo* (van der Eerden et al., 2005). Chamoux et al. reported that TRPV5 mediates the RANKL-induced sustained Ca^2+^ influx in more differentiated human osteoclasts *in vitro*, and surprisingly, they observed that inhibition of *Trpv5* leads to increased resorption pit formation *in vitro*. Unfortunately, data concerning the effect of *Trpv5* inactivation on osteoclast differentiation itself is lacking in this study (Chamoux et al., 2010). Thus, TRPV5 is present at the ruffled border of resorbing osteoclasts, but its exact role in osteoclastic Ca^2+^ signaling, osteoclastogenesis, and bone resorption is still unclear (Figure 3).

In conclusion, TRPV5 mediates calcium reabsorption in the kidney, and is hereby important for the maintenance of systemic calcium and bone homeostasis. In addition, TRPV5 is expressed in osteoclasts and may directly regulate osteoclast differentiation and/or functioning.

### TRPV4

That TRPV4 has a profound role in bone homeostasis is well demonstrated by the skeletal defects resulting from TRPV4 mutations in humans. Activating missense mutations in TRPV4 lead to a wide spectrum of autosomal-dominant skeletal dysplasias ranging from mild to lethal forms, and are characterized by cartilage and mineralization defects (Rock et al., 2008; Krakow et al., 2009). This finding, together with the observation that TRPV4 is expressed in chondrocytes, osteoclasts, and osteoblasts, suggests that TRPV4 directly controls the functioning of skeletal cells.

We showed that TRPV4 is present on the basolateral membrane of osteoclasts and is required for osteoclast differentiation by regulating intracellular Ca^2+^ signaling (see Ca^2+^ Signaling in Osteoclasts; Figure 3). More specifically, TRPV4 mediates the sustained Ca^2+^ influx that occurs in the late stages of osteoclast differentiation, when the Ca^2+^ oscillations are no longer present (Masuyama et al., 2008; Negishi-Koga and Takayanagi, 2009). This TRPV4-mediated Ca^2+^ influx secures intracellular Ca^2+^ concentrations, ensures NFATc1-regulated gene transcription, and regulates the terminal differentiation and activity of the osteoclasts. Indeed, intracellular Ca^2+^ levels, NFATc1 activity, osteoclast differentiation and resorptive capacity are reduced in *Trpv4^−/−^* osteoclasts. No difference in the percentage of oscillating cells, the frequency or the amplitude of the Ca^2+^ oscillations is observed between *Trpv4^−/−^* and *Trpv4*^+/+^ osteoclasts, implying that TRPV4 is not involved in the generation or the maintenance of the Ca^2+^ oscillations. In agreement with the *in vitro* findings, bone mass is increased in adult *Trpv4^−/−^* mice, and this is accompanied by a reduction in osteoclast abundance and bone resorption. Of note, TRPV4 inactivity does not affect systemic calcium homeostasis nor bone formation (Masuyama et al., 2008). Thus, Ca^2+^ oscillations and sustained Ca^2+^ influx via TRPV4 are sequentially required for osteoclast differentiation. In agreement with these finding, *Trpv4^−/−^* mice are also protected against unloading-induced bone loss (Mizoguchi et al., 2008). In addition, a recent study shows that activation of TRPV4 specifically in osteoclasts increased osteoclast differentiation, which leads to bone loss. The authors also demonstrate that TRPV4 not only affects osteoclast differentiation via the Ca^2+^-mediated activation of NFATc1, but that TRPV4 also influences osteoclast migration and fusion via Ca^2+^/calmodulin-mediated effects on myosin IIa (Masuyama et al., 2012). Thus, TRPV4 is critically involved in osteoclast differentiation and migration, by mediating the sustained Ca^2+^ influx in more differentiated osteoclasts.

TRPV4 is also expressed in chondrocytes, where it modulates differentiation and osmotically induced Ca^2+^ signaling (see Ca^2+^ Signaling in Chondrocytes; Figure 5). First of all, TRPV4 activation is found to induce SOX9 expression, a master regulator of chondrogenesis (Bi et al., 1999) through a Ca^2+^/calmodulin pathway, and by doing so, TRPV4 potentiates chondrogenic differentiation *in vitro* (Muramatsu et al., 2007). This finding indicates that TRPV4 is positively involved in chondrogenesis. Secondly, TRPV4 mediates the chondrocyte response to hypo-osmotic stress, which is required to maintain joint health. Indeed, suppression of TRPV4 activity abolishes the osmotic sensitivity of chondrocytes, impairs the increase in intracellular Ca^2+^ levels, and suppresses the volume decrease (Phan et al., 2009). Chemical disruption of the primary cilium also eliminates the Ca^2+^ signaling in response to an activator of TRPV channels and hypo-osmotic stimuli (Phan et al., 2009). This finding suggests that an intact cilium is required for TRPV4-mediated Ca^2+^ signaling. Remarkably, TRPV4 is distributed all over the plasma membrane, and was thus not strictly localized to the cilium (Phan et al., 2009). Consistent with these *in vitro* findings, *Trpv4^−/−^* mice develop early and severe osteoarthritis with progressive calcification of the joint tissue (Clark et al., 2010). Altogether, TRPV4 has a chondroprotective role by mediating the response to hypo-osmotic stimuli.

Thus, TRPV4 directly regulates differentiation and functioning of osteoclasts and chondrocytes by mediating intracellular Ca^2+^ influx and signaling.

### TRPV2

TRPV2 is expressed in osteoclasts and is likely involved in the Ca^2+^-permeable pathway that mediates osteoclastic Ca^2+^ oscillations (see Ca^2+^ Signaling in Osteoclasts; Figure 3). In fact, inhibition of TRPV2 hampers the RANKL-induced Ca^2+^ oscillations, NFATc1 activation, and osteoclastogenesis *in vitro* (Kajiya et al., 2010), yet conclusive *in vivo* evidence is currently lacking.

### TRPV1

TRPV1 is expressed in osteoblasts and osteoclasts and promotes the differentiation of both cell types. Indeed, pharmacological blockage of TRPV1 inhibits *in vitro* osteoclast and osteoblast differentiation. Moreover, *in vivo* suppression of TRPV1 activity protects mice against ovariectomy-induced bone loss by reducing the increase in bone resorption and formation (Idris et al., 2010). Thus, TRPV1 may directly affect osteogenic cell differentiation.

More importantly, TRPV1 is present in peripheral neurons that innervate bone, and TRPV1-mediated Ca^2+^ signaling in these neurons has an important function in the pain sensation that often accompanies bone metastases or inflammatory osteoarthritis. Indeed, growing tumor cells trigger osteoclasts to resorb bone excessively and this process induces an acid microenvironment due to the secretion of H^+^ by the osteoclasts. This acidity activates TRPV1 and other acid-sensing ion channels on the nociceptors that innervate bone. The associated Ca^2+^ influx triggers a series of events that are associated with pain (Nakanishi et al., 2010). Consequently, suppression of the excessive bone resorption or TRPV1 activity significantly lowers bone pain in mice (Nagae et al., 2006, 2007; Tong et al., 2010).

## Conclusion

Bone homeostasis depends on the external calcium balance as well as on intracellular Ca^2+^ signaling that regulates bone cell functioning and differentiation. A number of TRP channels are critically involved in the regulation of these processes, including intestinal calcium absorption (TRPV6), renal calcium reabsorption (TRPV5), osteoclastogenesis (TRPV4, TRPV5, TRPV2, TRPV1), osteoblastogenesis (TRPV1), chondrocyte differentiation/functioning (TRPV4), and bone pain sensation (TRPV1). The combination of *in vitro* and *in vivo* studies have provided tremendous insight in the role of TRP channels in calcium and bone homeostasis, but some aspects still require further investigation. First of all, the finding that TRPV6 is not essential for active intestinal transport, urges the search for new Ca^2+^ channels or alternative mechanisms that mediate calcium transport in the intestine. Secondly, the mechanisms that modulate Ca^2+^ oscillations during the early of osteoclastogenesis are still largely unknown, yet of interest given that this pathway is crucial for the autoamplification of NFATc1 and thus osteoclast differentiation. Thirdly, despite the fact that NFAT is important for osteoblast differentiation, the Ca^2+^ entry mechanisms that evoke NFAT activation are largely unexplored. In a next step, *in vitro* studies combined with the generation of mouse models that target the different TRP channels in a cell-specific manner, will provide more insight in their role in calcium and bone homeostasis. A better understanding is required to define whether the different TRP channels may serve as therapeutic targets in conditions of bone loss. Indeed one may speculate to use TRPV5 and TRPV6 agonists to maximize the calcium availability for bone, TRPV4 modulators to suppress osteoclast differentiation or to treat genetic dysfunctioning of TRPV4, etc. Thorough insight may also help to avoid possible side-effects that may occur when inhibiting/stimulating their function since TRPV channels are structurally related and thus require the development of highly specific modulators. In addition, the different TRPV channels have numerous important physiological functions in different tissues, a condition that may hinder its general application. To date, only TRPV1 modulators are used in the clinic as part of analgesic therapy (Moran et al., 2011).

## Conflict of Interest Statement

The authors declare that the research was conducted in the absence of any commercial or financial relationships that could be construed as a potential conflict of interest.

## References

[B1] Aliprantis A. O. Ueki Y. Sulyanto R. Park A. Sigrist K. S. Sharma S. M. Ostrowski M. C. Olsen B. R. Glimcher L. H. (2008). NFATc1 in mice represses osteoprotegerin during osteoclastogenesis and dissociates systemic osteopenia from inflammation in cherubism. J. Clin. Invest. 118, 3775–3789 10.1172/JCI35711 18846253 PMC2564610

[B2] Benn B. S. Ajibade D. Porta A. Dhawan P. Hediger M. Peng J. B. Jiang Y. Oh G. T. Jeung E. B. Lieben L. Bouillon R. Carmeliet G. Christakos S. (2008). Active intestinal calcium transport in the absence of transient receptor potential vanilloid type 6 and calbindin-D9k. Endocrinology 149, 3196–3205 10.1210/en.2007-1655 18325990 PMC2408805

[B3] Bi W. Deng J. M. Zhang Z. Behringer R. R. de C. B. (1999). Sox9 is required for cartilage formation. Nat. Genet. 22, 85–89 10.1038/8792 10319868

[B4] Bianco S. D. Peng J. B. Takanaga H. Suzuki Y. Crescenzi A. Kos C. H. Zhuang L. Freeman M. R. Gouveia C. H. Wu J. Luo H. Mauro T. Brown E. M. Hediger M. A. (2007). Marked disturbance of calcium homeostasis in mice with targeted disruption of the Trpv6 calcium channel gene. J. Bone Miner. Res. 22, 274–285 10.1359/jbmr.061110 17129178 PMC4548943

[B5] Bikle D. D. (2007). Vitamin D insufficiency/deficiency in gastrointestinal disorders. J. Bone Miner. Res. 22(Suppl. 2), V50–V54 10.1359/jbmr.07s208 18290722

[B6] Blair H. C. Schlesinger P. H. Huang C. L. Zaidi M. (2007). Calcium signalling and calcium transport in bone disease. Subcell. Biochem. 45, 539–562 10.1007/978-1-4020-6191-2_21 18193652 PMC2970924

[B7] Bouillon R. Carmeliet G. Verlinden L. van Etten E. Verstuyf A. Luderer H. F. Lieben L. Mathieu C. Demay M. (2008). Vitamin D and human health: lessons from vitamin D receptor null mice. Endocr. Rev. 29, 726–776 10.1210/er.2008-0004 18694980 PMC2583388

[B8] Bullamore J. R. Wilkinson R. Gallagher J. C. Nordin B. E. Marshall D. H. (1970). Effect of age on calcium absorption. Lancet 2, 535–537 10.1016/S0140-6736(70)91344-9 4195202

[B9] Chamoux E. Bisson M. Payet M. D. Roux S. (2010). TRPV-5 mediates a receptor activator of NF-kappaB (RANK) ligand-induced increase in cytosolic Ca2+ in human osteoclasts and down-regulates bone resorption. J. Biol. Chem. 285, 25354–25362 10.1074/jbc.M109.075234 20547482 PMC2919098

[B10] Clark A. L. Votta B. J. Kumar S. Liedtke W. Guilak F. (2010). Chondroprotective role of the osmotically sensitive ion channel transient receptor potential vanilloid 4: age- and sex-dependent progression of osteoarthritis in Trpv4-deficient mice. Arthritis Rheum. 62, 2973–2983 10.1002/art.27624 20583100 PMC3027356

[B11] Guilak F. Leddy H. A. Liedtke W. (2010). Transient receptor potential vanilloid 4: the sixth sense of the musculoskeletal system? Ann. N. Y. Acad. Sci. 1192, 404–409 10.1111/j.1749-6632.2010.05389.x 20392266 PMC3580043

[B12] Hoenderop J. G. Nilius B. Bindels R. J. (2005). Calcium absorption across epithelia. Physiol. Rev. 85, 373–422 10.1152/physrev.00003.2004 15618484

[B13] Hoenderop J. G. van Leeuwen J. P. van der Eerden B. C. Kersten F. F. van der Kemp A. W. Merillat A. M. Waarsing J. H. Rossier B. C. Vallon V. Hummler E. Bindels R. J. (2003). Renal Ca2+ wasting, hyperabsorption, and reduced bone thickness in mice lacking TRPV5. J. Clin. Invest. 112, 1906–1914 10.1172/JCI19826 14679186 PMC297001

[B14] Hwang S. Y. Putney J. W. (2011). Orai1-mediated calcium entry plays a critical role in osteoclast differentiation and function by regulating activation of the transcription factor NFATc1. FASEB J. 26, 1484–1492 10.1096/fj.11-194399 22198385 PMC3316896

[B15] Idris A. I. Landao-Bassonga E. Ralston S. H. (2010). The TRPV1 ion channel antagonist capsazepine inhibits osteoclast and osteoblast differentiation in vitro and ovariectomy induced bone loss in vivo. Bone 46, 1089–1099 10.1016/j.bone.2010.01.368 20096813

[B16] Kajiya H. Okamoto F. Nemoto T. Kimachi K. Toh-Goto K. Nakayana S. Okabe K. (2010). RANKL-induced TRPV2 expression regulates osteoclastogenesis via calcium oscillations. Cell Calcium 48, 260–269 10.1016/j.ceca.2010.09.010 20980052

[B17] Koga T. Inui M. Inoue K. Kim S. Suematsu A. Kobayashi E. Iwata T. Ohnishi H. Matozaki T. Kodama T. Taniguchi T. Takayanagi H. Takai T. (2004). Costimulatory signals mediated by the ITAM motif cooperate with RANKL for bone homeostasis. Nature 428, 758–763 10.1038/nature02444 15085135

[B18] Koga T. Matsui Y. Asagiri M. Kodama T. de C. B. Nakashima K. Takayanagi H. (2005). NFAT and Osterix cooperatively regulate bone formation. Nat. Med. 11, 880–885 10.1038/nm1270 16041384

[B19] Krakow D. Vriens J. Camacho N. Luong P. Deixler H. Funari T. L. Bacino C. A. Irons M. B. Holm I. A. Sadler L. Okenfuss E. B. Janssens A. Voets T. Rimoin D. L. Lachman R. S. Nilius B. Cohn D. H. (2009). Mutations in the gene encoding the calcium-permeable ion channel TRPV4 produce spondylometaphyseal dysplasia, Kozlowski type and metatropic dysplasia. Am. J. Hum. Genet. 84, 307–315 10.1016/j.ajhg.2009.01.021 19232556 PMC2667978

[B20] Kuroda Y. Hisatsune C. Nakamura T. Matsuo K. Mikoshiba K. (2008). Osteoblasts induce Ca2+ oscillation-independent NFATc1 activation during osteoclastogenesis. Proc. Natl. Acad. Sci. U.S.A 105, 8643–8648 10.1073/pnas.0800642105 18552177 PMC2438406

[B21] Kutuzova G. D. Sundersingh F. Vaughan J. Tadi B. P. Ansay S. E. Christakos S. DeLuca H. F. (2008). TRPV6 is not required for 1alpha,25-dihydroxyvitamin D3-induced intestinal calcium absorption in vivo. Proc. Natl. Acad. Sci. U.S.A. 105, 19655–19659 10.1073/pnas.0810761105 19073913 PMC2605002

[B22] Lieben L. Benn B. S. Ajibade D. Stockmans I. Moermans K. Hediger M. A. Peng J. B. Christakos S. Bouillon R. Carmeliet G. (2010). Trpv6 mediates intestinal calcium absorption during calcium restriction and contributes to bone homeostasis. Bone 47, 301–308 10.1016/j.bone.2010.04.595 20399919 PMC2902603

[B23] Lieben L. Carmeliet G. Masuyama R. (2011). Calcemic actions of vitamin D: effects on the intestine, kidney and bone. Best Pract. Res. Clin. Endocrinol. Metab. 25, 561–572 10.1016/j.beem.2011.05.008 21872798

[B24] Lieben L. Masuyama R. Torrekens S. Van L. R. Schrooten J. Baatsen P. Lafage-Proust M. H. Dresselaers T. Feng J. Q. Bonewald L. F. Meyer M. B. Pike J. W. Bouillon R. Carmeliet G. (2012). Normocalcemia is maintained in mice under conditions of calcium malabsorption by vitamin D-induced inhibition of bone mineralization. J. Clin. Invest. 122, 1803–1815 10.1172/JCI45890 22523068 PMC3336970

[B25] Little R. Muimo R. Robson L. Harris K. Grabowski P. S. (2011). The transient receptor potential ion channel TRPV6 is expressed at low levels in osteoblasts and has little role in osteoblast calcium uptake. PLoS ONE 6, e28166 10.1371/journal.pone.0028166 22163264 PMC3226639

[B26] Lu X. L. Huo B. Chiang V. Guo X. E. (2012). Osteocytic network is more responsive in calcium signaling than osteoblastic network under fluid flow. J. Bone Miner. Res. 27, 563–574 10.1002/jbmr.1474 22113822 PMC3343217

[B27] Mancilla E. E. Galindo M. Fertilio B. Herrera M. Salas K. Gatica H. Goecke A. (2007). L-type calcium channels in growth plate chondrocytes participate in endochondral ossification. J. Cell. Biochem. 101, 389–398 10.1002/jcb.21183 17243114

[B28] Masuyama R. Mizuno A. Komori H. Kajiya H. Uekawa A. Kitaura H. Okabe K. Ohyama K. Komori T. (2012). Calcium/calmodulin-signaling supports TRPV4 activation in osteoclasts and regulates bone mass. J. Bone Miner. Res. 27, 1708–1721 10.1002/jbmr.1629 22492541

[B29] Masuyama R. Vriens J. Voets T. Karashima Y. Owsianik G. Vennekens R. Lieben L. Torrekens S. Moermans K. Vanden Bosch A. Bouillon R. Nilius B. Carmeliet G. (2008). TRPV4-mediated calcium influx regulates terminal differentiation of osteoclasts. Cell Metab. 8, 257–265 10.1016/j.cmet.2008.08.002 18762026

[B30] Mizoguchi F. Mizuno A. Hayata T. Nakashima K. Heller S. Ushida T. Sokabe M. Miyasaka N. Suzuki M. Ezura Y. Noda M. (2008). Transient receptor potential vanilloid 4 deficiency suppresses unloading-induced bone loss. J. Cell. Physiol. 216, 47–53 10.1002/jcp.21374 18264976

[B31] Moran M. M. McAlexander M. A. Biro T. Szallasi A. (2011). Transient receptor potential channels as therapeutic targets. Nat. Rev. Drug Discov. 10, 601–620 10.1038/nrd3456 21804597

[B32] Muramatsu S. Wakabayashi M. Ohno T. Amano K. Ooishi R. Sugahara T. Shiojiri S. Tashiro K. Suzuki Y. Nishimura R. Kuhara S. Sugano S. Yoneda T. Matsuda A. (2007). Functional gene screening system identified TRPV4 as a regulator of chondrogenic differentiation. J. Biol. Chem. 282, 32158–32167 10.1074/jbc.M706158200 17804410

[B33] Nagae M. Hiraga T. Wakabayashi H. Wang L. Iwata K. Yoneda T. (2006). Osteoclasts play a part in pain due to the inflammation adjacent to bone. Bone 39, 1107–1115 10.1016/j.bone.2006.04.033 16769263

[B34] Nagae M. Hiraga T. Yoneda T. (2007). Acidic microenvironment created by osteoclasts causes bone pain associated with tumor colonization. J. Bone Miner. Metab. 25, 99–104 10.1007/s00774-006-0734-8 17323179

[B35] Nakanishi M. Hata K. Nagayama T. Sakurai T. Nishisho T. Wakabayashi H. Hiraga T. Ebisu S. Yoneda T. (2010). Acid activation of Trpv1 leads to an up-regulation of calcitonin gene-related peptide expression in dorsal root ganglion neurons via the CaMK-CREB cascade: a potential mechanism of inflammatory pain. Mol. Biol. Cell 21, 2568–2577 10.1091/mbc.E10-01-0049 20534813 PMC2912344

[B36] Negishi-Koga T. Takayanagi H. (2009). Ca2+-NFATc1 signaling is an essential axis of osteoclast differentiation. Immunol. Rev. 231, 241–256 10.1111/j.1600-065X.2009.00821.x 19754901

[B37] Nilius B. Owsianik G. Voets T. Peters J. A. (2007). Transient receptor potential cation channels in disease. Physiol. Rev. 87, 165–217 10.1152/physrev.00021.2006 17237345

[B38] Pedersen S. F. Owsianik G. Nilius B. (2005). TRP channels: an overview. Cell Calcium 38, 233–252 10.1016/j.ceca.2005.06.028 16098585

[B39] Phan M. N. Leddy H. A. Votta B. J. Kumar S. Levy D. S. Lipshutz D. B. Lee S. H. Liedtke W. Guilak F. (2009). Functional characterization of TRPV4 as an osmotically sensitive ion channel in porcine articular chondrocytes. Arthritis Rheum. 60, 3028–3037 10.1002/art.24799 19790068 PMC2846816

[B40] Robinson L. J. Blair H. C. Barnett J. B. Zaidi M. Huang C. L. (2010). Regulation of bone turnover by calcium-regulated calcium channels. Ann. N. Y. Acad. Sci. 1192, 351–357 10.1111/j.1749-6632.2009.05219.x 20392259 PMC11610510

[B41] Robinson L. J. Mancarella S. Songsawad D. Tourkova I. L. Barnett J. B. Gill D. L. Soboloff J. Blair H. C. (2012). Gene disruption of the calcium channel Orai1 results in inhibition of osteoclast and osteoblast differentiation and impairs skeletal development. Lab. Invest. 92, 1071–1083 10.1038/labinvest.2012.72 22546867 PMC3387291

[B42] Rock M. J. Prenen J. Funari V. A. Funari T. L. Merriman B. Nelson S. F. Lachman R. S. Wilcox W. R. Reyno S. Quadrelli R. Vaglio A. Owsianik G. Janssens A. Voets T. Ikegawa S. Nagai T. Rimoin D. L. Nilius B. Cohn D. H. (2008). Gain-of-function mutations in TRPV4 cause autosomal dominant brachyolmia. Nat. Genet. 40, 999–1003 10.1038/ng.166 18587396 PMC3525077

[B43] Sato K. Suematsu A. Nakashima T. Takemoto-Kimura S. Aoki K. Morishita Y. Asahara H. Ohya K. Yamaguchi A. Takai T. Kodama T. Chatila T. A. Bito H. Takayanagi H. (2006). Regulation of osteoclast differentiation and function by the CaMK-CREB pathway. Nat. Med. 12, 1410–1416 10.1038/nm1515 17128269

[B44] Sun L. Blair H. C. Peng Y. Zaidi N. Adebanjo O. A. Wu X. B. Wu X. Y. Iqbal J. Epstein S. Abe E. Moonga B. S. Zaidi M. (2005). Calcineurin regulates bone formation by the osteoblast. Proc. Natl. Acad. Sci. U.S.A. 102, 17130–17135 10.1073/pnas.0403198102 16286645 PMC1288002

[B45] Takayanagi H. Kim S. Koga T. Nishina H. Isshiki M. Yoshida H. Saiura A. Isobe M. Yokochi T. Inoue J. Wagner E. F. Mak T. W. Kodama T. Taniguchi T. (2002). Induction and activation of the transcription factor NFATc1 (NFAT2) integrate RANKL signaling in terminal differentiation of osteoclasts. Dev. Cell 3, 889–901 10.1016/S1534-5807(02)00369-6 12479813

[B46] Taschner M. J. Rafigh M. Lampert F. Schnaiter S. Hartmann C. (2008). Ca2+/Calmodulin-dependent kinase II signaling causes skeletal overgrowth and premature chondrocyte maturation. Dev. Biol. 317, 132–146 10.1016/j.ydbio.2008.02.007 18342847

[B47] Tong Z. Luo W. Wang Y. Yang F. Han Y. Li H. Luo H. Duan B. Xu T. Maoying Q. Tan H. Wang J. Zhao H. Liu F. Wan Y. (2010). Tumor tissue-derived formaldehyde and acidic microenvironment synergistically induce bone cancer pain. PLoS ONE 5, e10234 10.1371/journal.pone.0010234 20422007 PMC2858155

[B48] Van Cromphaut S. J. Dewerchin M. Hoenderop J. G. Stockmans I. Van Herck E. Kato S. Bindels R. J. Collen D. Carmeliet P. Bouillon R. Carmeliet G. (2001). Duodenal calcium absorption in vitamin D receptor-knockout mice: functional and molecular aspects. Proc. Natl. Acad. Sci. U.S.A. 98, 13324–13329 10.1073/pnas.231474698 11687634 PMC60869

[B49] van der Eerden B. C. Hoenderop J. G. de Vries T. J. Schoenmaker T. Buurman C. J. Uitterlinden A. G. Pols H. A. Bindels R. J. van Leeuwen J. P. (2005). The epithelial Ca2+ channel TRPV5 is essential for proper osteoclastic bone resorption. Proc. Natl. Acad. Sci. U.S.A. 102, 17507–17512 10.1073/pnas.0505789102 16291808 PMC1297662

[B50] van der Eerden B. C. Weissgerber P. Fratzl-Zelman N. Olausson J. Hoenderop J. G. Schreuders-Koedam M. Eijken M. Roschger P. de Vries T. J. Chiba H. Klaushofer K. Flockerzi V. Bindels R. J. Freichel M. van Leeuwen J. P. (2012). The transient receptor potential channel TRPV6 is dynamically expressed in bone cells but is not crucial for bone mineralization in mice. J. Cell. Physiol. 227, 1951–1959 10.1002/jcp.22923 21732366

[B51] Winslow M. M. Pan M. Starbuck M. Gallo E. M. Deng L. Karsenty G. Crabtree G. R. (2006). Calcineurin/NFAT signaling in osteoblasts regulates bone mass. Dev. Cell 10, 771–782 10.1016/j.devcel.2006.04.006 16740479

[B52] Yeo H. Beck L. H. Thompson S. R. Farach-Carson M. C. McDonald J. M. Clemens T. L. Zayzafoon M. (2007). Conditional disruption of calcineurin B1 in osteoblasts increases bone formation and reduces bone resorption. J. Biol. Chem. 282, 35318–35327 10.1074/jbc.M705750200 17884821

